# Comparison of Two Posterior Minimally Invasive Approaches for Odontoid Fractures: Midline Nuchal Ligament Approach vs. Paramedian Muscle‐Splitting Approach

**DOI:** 10.1111/os.70213

**Published:** 2025-12-12

**Authors:** Youcai Qiu, Liang Wang, Yijin Wang, Yang Li, Xuhua Lu

**Affiliations:** ^1^ Department of Orthopaedics Shanghai Changzheng Hospital, Navy Medical University Shanghai China; ^2^ Department of Orthopaedics The 941th Hospital of Joint Logistics Support Force of Chinese People's Liberation Army China

**Keywords:** greater occipital nerve, midline nuchal ligament approach, minimally invasive approach, odontoid fractures, paramedian muscle‐splitting approach

## Abstract

**Objective:**

The posterior minimally invasive approaches for odontoid fractures include the midline nuchal ligament approach (MNLA) and the paramedian muscle‐splitting approach (PMSA). However comparative data on their anatomical characteristics and clinical efficacy remain scarce to date. The objective of this study is to determine the differences in anatomy and clinical outcomes between the MNLA and the PMSA for reduction and temporary internal fixation of odontoid fractures.

**Methods:**

This retrospective analysis focused on 31 patients with odontoid fractures from February 2021 to December 2023. Among them,16 patients underwent PMSA and 15 patients underwent MNLA. Various parameters were compared between the two groups, including operation time, intraoperative blood loss, postoperative complications, edema rates of cervical posterior muscles, the range of motion in rotation of C1–C2, patient satisfaction, Visual Analogue Scale score for neck pain, axial symptom scores, and neck disability index. Additionally, an anatomical study was performed; the PMSA and the MNLA were simulated on six fresh cadaveric specimens to compare the anatomical differences in surgical exposure between the two approaches.

**Results:**

In the clinical study, both groups successfully achieved fracture healing. Compared with the PMSA group, the MNLA group had several advantages, including shorter operative times, lower intraoperative blood loss, and a lower edema rate of posterior cervical muscles. However, similar results were observed between the two groups in terms of the range of motion in rotation of C1–C2, patient satisfaction, Visual Analogue Scale score for neck pain, axial symptom scores, and neck disability index at the last follow‐up. In the cadaveric study, we found the trapezius‐splenius capitis interface and the course of the greater occipital nerve (GON) varied significantly and the GON was present in the surgical field in 2 of 6 specimens in the PMSA, which brought difficulties for the surgical operation. In contrast, the MNLA, using the spinous process of C2 and the obliquus capitis inferior (OCI) as anatomical landmarks, provided a simpler surgical procedure and easier exposure.

**Conclusion:**

Both the MNLA and the PMSA demonstrated favorable clinical outcomes for the treatment of odontoid fractures. However, compared with the PMSA, the MNLA, using the spinous process of C2 and the OCI as anatomical landmarks, offers advantages of the stability of the surgical procedure, easy exposure, and reduced iatrogenic damage to the cervical posterior muscles and GON.

## Graphical abstract

The midline nuchal ligament approach yields clinical outcomes similar to those of the paramedian muscle‐splitting technique for odontoid fractures, while offering a more stable surgical procedure and reducing iatrogenic injury to the cervical posterior muscles and the greater occipital nerve.

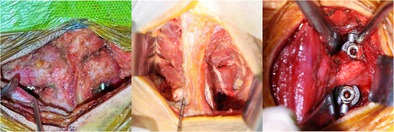

## 1 Introduction

Odontoid fractures are the most common C2 fractures, accounting for about 10%–20% of all cervical injuries [1]. Current treatment strategies remain controversial with no definitive consensus on optimal management. Anterior screw fixation is considered the preferred treatment for odontoid fractures with intact transverse ligament due to its preservation of atlantoaxial rotation. However, this technique is only indicated for Grauer type II_B_ fracture [2] extending from anterior‐superior inferior to posterior‐inferior. Posterior temporary fixation, followed by instrumentation removal after fracture union to restore atlantoaxial rotation, is regarded as a salvage option for patients with intact transverse ligament who have contraindications to or failure of anterior approaches [3]. Compared with atlantoaxial fusion techniques, this method demonstrates superior functional outcomes [4]. However, the traditional approach requires subperiosteal stripping of cervical posterior muscles, including the obliquus capitis inferior (OCI), semispinalis cervicis (SSCe), rectus capitis posterior minor (RCPmi), rectus capitis posterior major (RCPma), which are essential for the kinematics and stabilization of the atlantoaxial complex [5, 6].

The evolution of medical technology has led to the development of minimally invasive techniques. This approach usually utilizes natural intermuscular spaces for dissection and exposure, which helps to minimize iatrogenic damage to blood vessels, nerves, and muscles, ultimately reducing postoperative complications. Current minimally invasive techniques for posterior temporary fixation in the treatment of odontoid fractures predominantly involve the midline nuchal ligament approach (MNLA) [7, 8, 9] and the paramedian muscle‐splitting approach (PMSA) [10, 11, 12, 13]. The optimal selection between the two minimally invasive approaches remains controversial, with no definitive consensus in current clinical practice.

This research performed a retrospective comparative analysis of clinical and radiographic outcomes associated with the two approaches for treating odontoid fractures. Additionally, surgical simulations were conducted using fresh cadaveric specimens to identify anatomical differences. The results offer detailed anatomical and clinical insights to aid in the selection of minimally invasive approaches. Thus, the present study was conducted to (i) elucidate the anatomical differences between the MNLA and PMSA, and (ii) compare the clinical efficacy of these two approaches in the treatment of odontoid fractures.

## 2 Materials and Methods

### 2.1 Clinical Study

All procedures were performed in strict adherence to the ethical principles of the Declaration of Helsinki and approved by the Ethics Committee (approval number: 2022SLYS10) of the hospital. Written informed consent was obtained from all participants.

#### 2.1.1 Subjects

The inclusion criteria were as follows: (1) Grauer type IIC fractures [2] that manifested with anterior inferior to posterior superior or comminuted fractures; (2) Grauer type IIA and IIB fractures [2] that were not amenable to anterior screw fixation; (3) Grauer type III fractures [2] that could not tolerate prolonged external fixation; (4) patients aged 18–60 years with complete clinical follow‐up data; (5) postoperative follow‐up was at least 1 year.

The exclusion criteria were as follows: (1) odontoid fractures accompanied by atlantoaxial instability, disruption of the transverse ligament, or atlantoaxial subluxation with atlanto‐dental interval > 3 mm necessitate the performance of posterior C1–C2 fusion; (2) inadequate physical condition or significant polytrauma, resulting in an inability to endure the second surgery; (3) history of anterior or posterior cervical surgery; (4) history of cervical vertebrae deformity.

#### 2.1.2 Patient Information

From February 2021 to August 2022, we performed the posterior temporary fixation using the PMSA, and from September 2022 to December 2023, the surgical protocol transitioned to the MNLA. Consequently, 31 consecutive patients meeting inclusion criteria were stratified into two groups: PMSA group (*n* = 16) and MNLA group (*n* = 15).

#### 2.1.3 Surgical Procedure

All patients underwent nasotracheal or fiberoptic endotracheal intubation. After the induction of general anesthesia, the patients were placed in a prone stance on a specially designed plaster bed. Continuous skull traction was employed during the surgical procedure to facilitate the reduction of the fracture. C‐arm fluoroscopy was utilized to monitor and confirm the successful reduction of the fracture. The occipital region was disinfected and covered with a sterile sheet, and a skin protection film was applied. A midline incision was made from the external occipital protuberance to the spinous process of C2.

**FIGURE 1:**
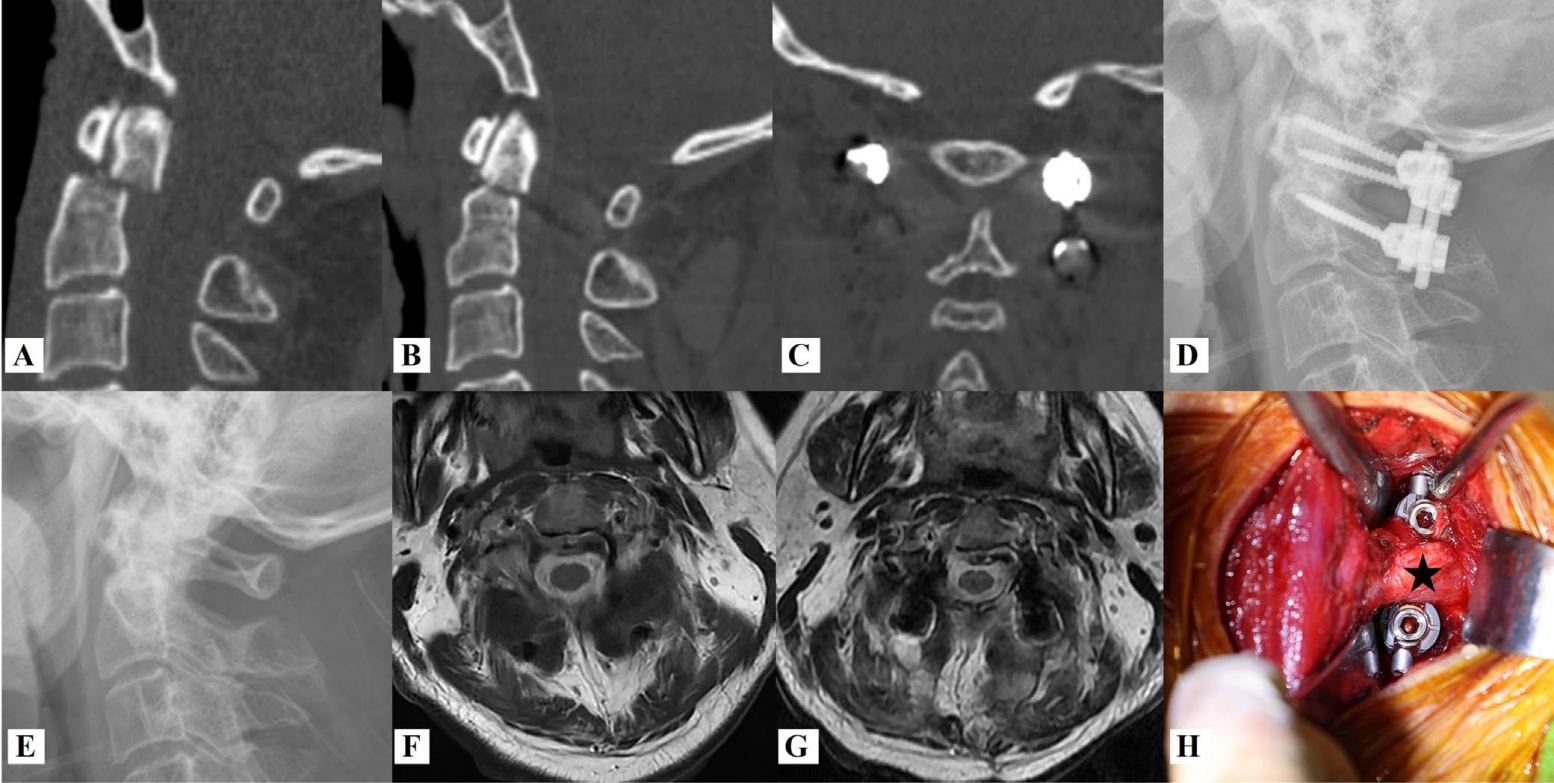
Male, 45 years old, injured in fall accident, Grauer type II_A_ fracture treated by the PMSA. (A) Preoperative sagittal view of CT scan shows odontoid fracture combined with dislocation; (B) postoperative CT scan shows fracture was reset; (C) postoperative coronal view of CT scan shows the preserved signal of the muscles connected to the spinous process of C2; (D) postoperative lateral X‐ray image indicates that the fracture has been successfully realigned; (E) lateral X‐ray imaging of the upper cervical spine following the removal of the internal fixator revealed signs of odontoid healing; (F, G) axial views of MRI before surgery and 3rd days post‐operation; (H) posterior view of the surgical site after locking the screw‐rod system, the black star indicates the OCI.

PMSA group: The trapezius (Traps) muscle was first exposed, after which the space between the Traps and splenius capitis (SpCa) muscles was identified. Then the deep semispinalis capitis (SSCa) muscle was exposed, Blunt dissection through SSCa then revealed the suboccipital fat pad, allowing exposure of the deep muscles, namely RCPma, OCI and SSCe. The posterior arch of C1 was situated between RCPma and OCI, while the lamina of C2 was positioned between OCI and SSCe. The C1 lateral mass and C2 pedicle screws were inserted using the Harms and Melcher free‐hand technique [14]. The titanium rods were inserted below the OCI. After confirming the anatomical alignment of the fracture using intraoperative fluoroscopy, the screw‐rod system was locked. The operative area was flushed, and the incision was closed in layers without the use of a suction drainage tube. Figure 1 shows typical patients.

**FIGURE 2:**
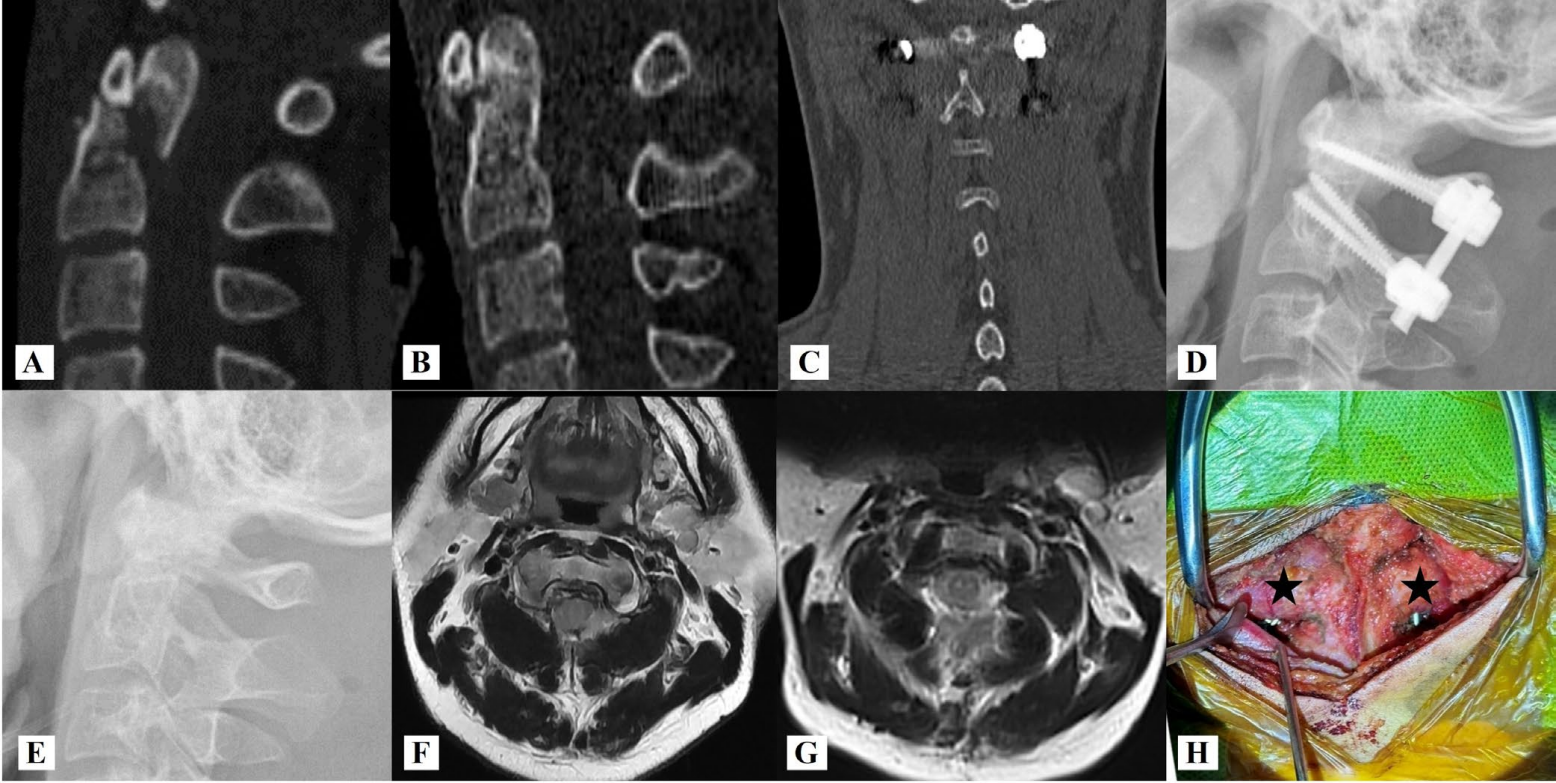
Male, 25 years old, injured in car accident, Grauer type II_B_ fracture treated by the MNLA. (A) Preoperative sagittal view of CT scan shows odontoid fracture combined with dislocation; (B) postoperative CT scan shows fracture was reset; (C) postoperative coronal view of CT scan shows the preserved signal of the muscles connected to the spinous process of C2; (D) postoperative lateral X‐ray image indicates that the fracture has been successfully realigned; (E) lateral X‐ray imaging of the upper cervical spine following the removal of the internal fixator revealed signs of odontoid healing; (F, G) axial views of MRI before surgery and 3rd days post‐operation; (H) posterior view of the surgical site after locking the screw‐rod system, the black star indicates the OCI.

MNLA group: The nuchal ligament was initially exposed and incised along the midline, after which the muscles connected to the spinous process of C2, specifically the RCPma, OCI and SSCe, were identified. Subsequently, the procedure was performed utilizing the same method as the PMSA. Figure 2 shows typical patients.

#### 2.1.4 Postoperative Treatment

The postoperative collar period was 6–8 weeks. Early mobilization with cervical collar support was encouraged following recovery from general anesthesia. Cervical CT scans and image reconstruction were conducted at least 3 months postoperatively or later to assess the healing of fractures. Once fracture healing was observed, the removal of instrumentation was performed through the identical surgical approach.

#### 2.1.5 Observation Indicators

X‐ray images were reviewed before surgery, and at 3, 6 and 9 months after the first surgery and after instrumentation removal. The stability of the upper cervical complex was evaluated on flexion‐extension lateral X‐ray images. The observation indicators after the first surgery and the removal of instrumentation were listed below.

**FIGURE 3:**
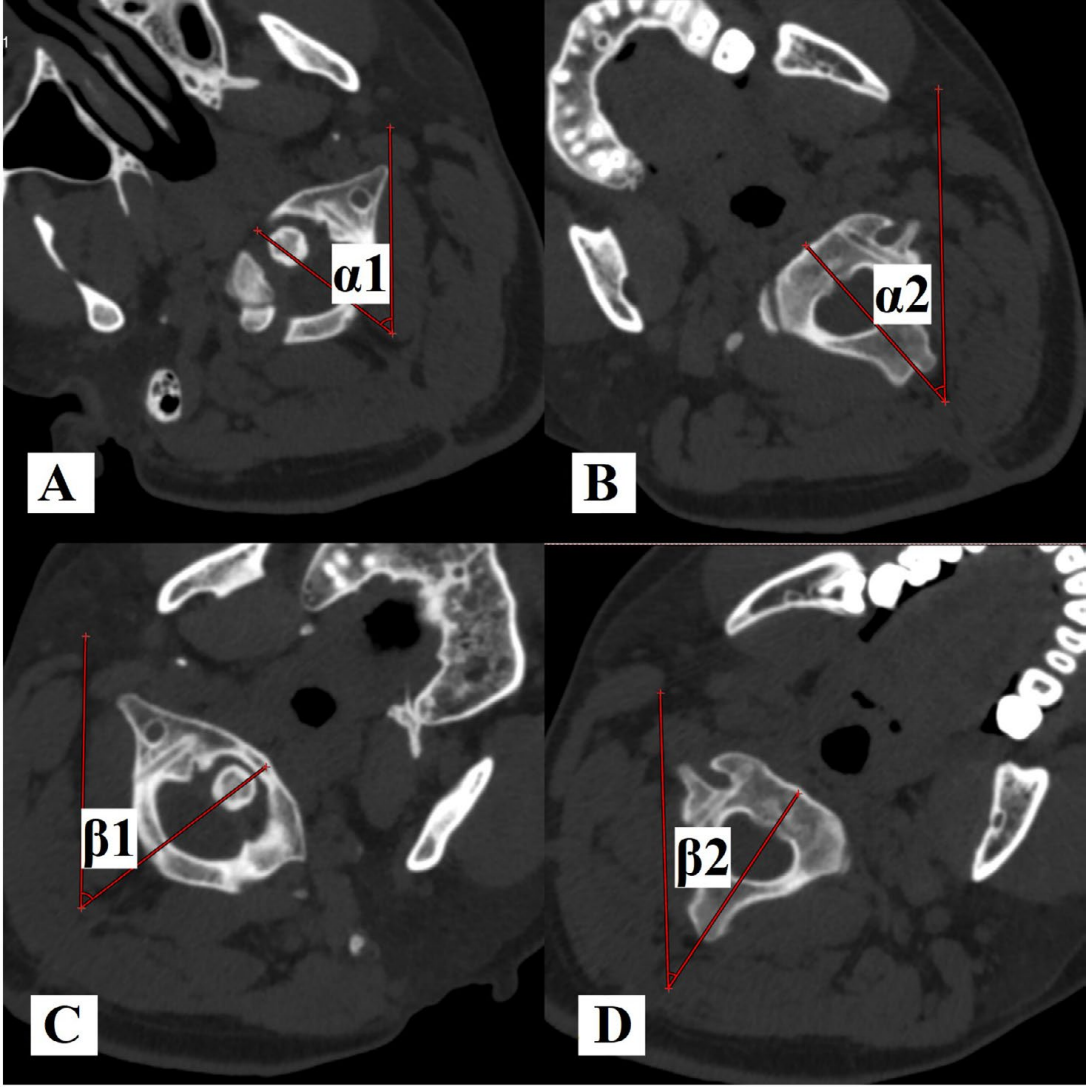
Measurements of ROM in rotation of C1–C2 on rotational CT scan, left side, α1–α2 = 15°, right side, β1–β2 = 20°.

Measurements of the range of motion (ROM) in rotation of C1–C2: Cervical rotational CT was utilized to evaluate ROM in the rotation of the C1–C2 in patients with the removal of instrumentation at the final follow‐up. The measurements were conducted using bone algorithms on the cervical rotational CT scan according to previous studies [3, 4, 15] (Figure 3).

**FIGURE 4:**
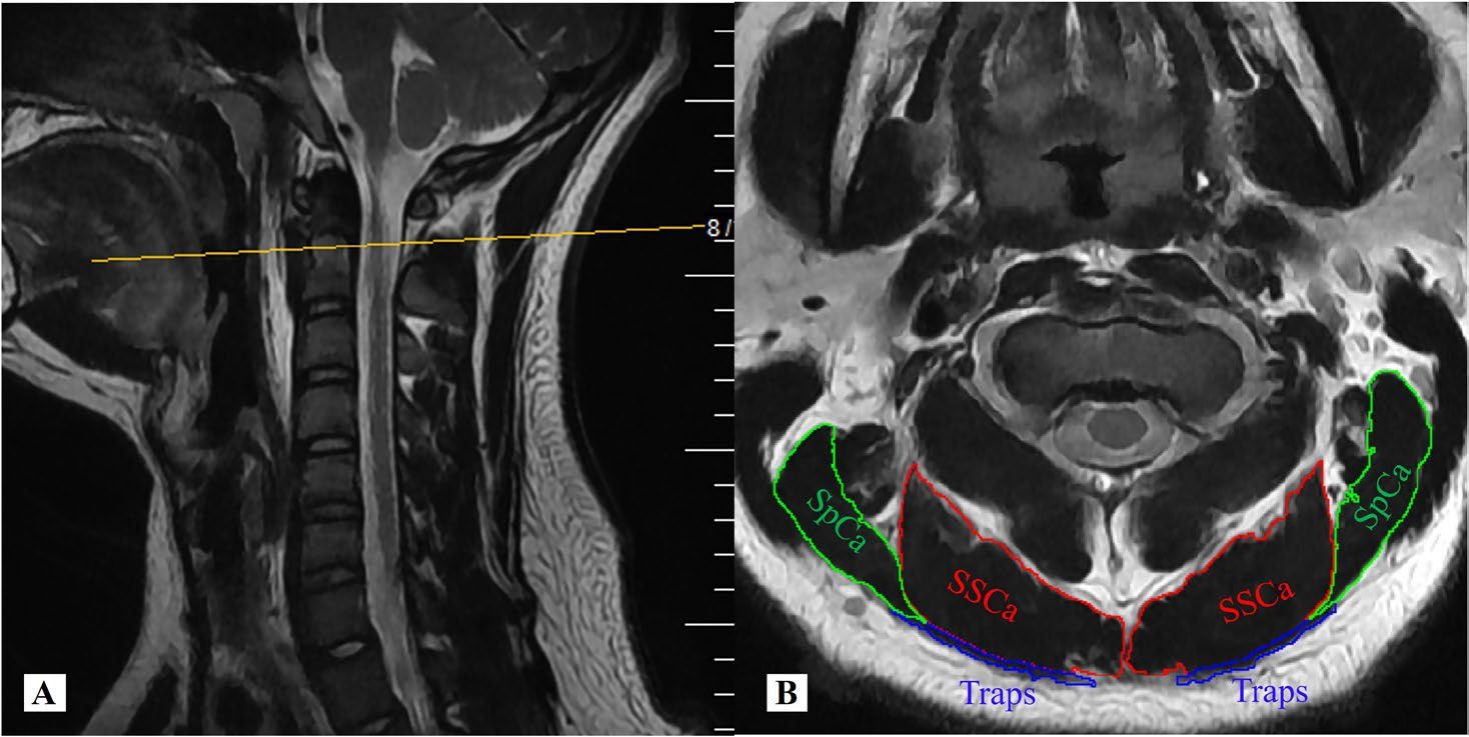
Schematic representation of the CSA of the cervical posterior muscles, include Traps (blue), SpCa (green), and SSCa (red).

Measurements of the cross‐sectional area (CSA) of the cervical posterior muscles at the C1–2 levels: The cervical spine was examined by magnetic resonance imaging (MRI) before surgery and 3 days post‐operation [16]. The CSA of the cervical posterior muscles, including Traps, SpCa and SSCa, was employed to assess the impact of surgical techniques on muscle injury. The CSA at the C1–2 levels was measured for evaluation on T2‐weighted sequences of MRI (Figure 4). The measurement was performed using ImageJ imaging software (version 1.54P, National Institutes of Health, Bethesda, Maryland, USA). The rate of muscle edema was calculated using the following formula: edema rate (%) = (postoperative CSA/preoperative CSA – 1) * 100%.

All radiographic measurements were performed by two independent researchers who were blinded to the surgical procedures. Each parameter was measured in triplicate to calculate the mean value.

The functional outcomes assessed through patient satisfaction [17], Visual Analogue Scale Score for Neck Pain (VASSNP) [18], Axial Symptom Scores (ASS) [19] and Neck Disability Index (NDI) [20] were compared between two groups at the final follow‐up.

#### 2.1.6 Statistical Analysis

All data were analyzed using SPSS Statistics version 29.0 (IBM Corp, Armonk, NY, USA). Continuous variables are expressed as mean ± standard deviation (*X ± S*). The data across the groups were examined using an independent samples *t*‐test. Categorical variables are presented as frequencies (*n*), analyzed by Fisher's exact test. Statistical significance was established at a threshold of *p* < 0.05.

### 2.2 Cadaver Study

Six fresh‐frozen adult cadaveric specimens (3 males, 3 females) were utilized in this anatomical study. Surgical simulations were performed bilaterally: the PMSA through dissecting the Traps and SpCa on one side, while employing the MNLA on the opposite side. The study protocol adhered to the Declaration of Helsinki principles and complied with the institutional and ethical standards of the School of Medicine. All specimens were screened to exclude prior cervical surgical interventions or congenital deformities.

The surgical procedure was conducted with each specimen in the prone position. The two approaches described above were utilized to expose C1 lateral mass and C2 pedicle respectively, with observation of differences in exposure processes between the two approaches. All procedures were performed by two surgeons. The identification of the nerves and muscles associated with the two methods was carried out by a professor of anatomy and a professor of surgery.

## 3 Results

### 3.1 Clinical Study

#### 3.1.1 General Information

Both groups successfully achieved fracture healing in postoperative 6 months. The intergroup differences in age, gender, BMI, fracture classification, cause of fracture, removal instrumentation time, and follow‐up time were not statistically significant (*p* > 0.05). The MNLA group demonstrated statistically significant advantages in operative time (94.19 ± 8.82 min vs. 68.07 ± 6.36 min, *p* < 0.001) and intraoperative blood loss (33.75 ± 8.06 mL vs. 25.33 ± 5.16 mL, *p* = 0.002) compared with the PMSA group (Table 1).

**TABLE 1:** Patient general information.

Variable	PMSA group	MNLA group	*p*
Age (*X ± S*, years)	47.94 ± 12.04	46.40 ± 14.07	0.746^a^
Sex (*n*)			0.563^b^
Male	11	10	
Female	5	5	
BMI (*X ± S*, kg/m^2^)	24.12 ± 2.98	24.72 ± 2.75	0.564^a^
Fracture classification (*n*)			0.742^b^
Type II_A_	0	1	
Type II_B_	8	8	
Type II_C_	5	4	
Type III	3	2	
Cause of fracture (*n*)			0.814^b^
Low‐energy fall	5	6	
Fall from a considerable height	3	2	
Motor vehicle accident	8	7	
Operation time (*X ± S*, min)	94.19 ± 8.82	68.07 ± 6.36	< 0.001^a^
Intraoperative blood loss (*X ± S*, mL)	33.75 ± 8.06	25.33 ± 5.16	0.002^a^
Removal instrumentation time (*X ± S*, months)	5.44 ± 0.51	5.27 ± 0.46	0.175^a^
Follow‐up time (*X ± S*, months)	19.25 ± 3.64	17.87 ± 2.10	0.209^a^

Abbreviations: BMI, body mass index; MNLA, midline nuchal ligament approach; PMSA, paramedian muscle‐splitting approach.

^a^ Independent samples *t*‐test.

^b^ Fisher's exact test.

#### 3.1.2 Observation Indicators Result

At final follow‐up, the total ROM showed no significant intergroup difference (38.94° ± 4.48° vs. 38.67° ± 6.03°, *p* = 0.870), with no radiographic evidence of upper cervical instability. The edema rate of cervical posterior muscles between two groups was compared at 3rd days post‐operation (Table 2). The MNLA group demonstrated statistically significant advantages in edema rate (Traps: 40.34% ± 5.27% vs. 9.76% ± 1.54%, *p* < 0.001; SpCa: 44.08% ± 4.10% vs. 10.75% ± 1.75%, *p* < 0.001; SSCa: 42.70% ± 5.00% vs. 30.70% ± 4.63%, *p* < 0.001) compared with the PMSA group.

**TABLE 2:** Edema rate of cervical posterior muscles between two groups.

		PMSA group	MNLA group	*p*
Traps	Pre‐operation CSA (*X ± S*, cm^2^)	1.17 ± 0.24	1.26 ± 0.18	0.238
	3rd days post‐operation CSA (*X ± S*, cm^2^)	1.63 ± 0.28	1.38 ± 0.18	0.007
	Edema rate (*X ± S*, %)	40.34 ± 5.27	9.76 ± 1.54	< 0.001
SpCa	Pre‐operation CSA (*X ± S*, cm^2^)	5.26 ± 0.45	5.42 ± 0.50	0.364
	3rd days post‐operation CSA (*X ± S*, cm^2^)	7.57 ± 0.48	5.99 ± 0.49	< 0.001
	Edema rate (*X ± S*, %)	44.08 ± 4.10	10.75 ± 1.75	< 0.001
SSCa	Pre‐operation CSA (*X ± S*, cm^2^)	12.44 ± 1.58	12.78 ± 2.12	0.618
	3rd days post‐operation CSA (*X ± S*, cm^2^)	17.68 ± 1.75	16.61 ± 2.16	0.137
	Edema rate (*X ± S*, %)	42.70 ± 5.00	30.70 ± 4.63	< 0.001

Abbreviations: CSA, cross‐sectional area; MNLA, midline nuchal ligament approach; SpCa, splenius capitis; SSCa, semispinalis capitis; PMSA, paramedian muscle‐splitting approach; Traps, trapezius.

The functional outcomes of the two groups were assessed at the final follow‐up (Table 3). No significant differences between the two groups were observed in the outcomes evaluated by patient satisfaction, VASSNP, ASS, and NDI.

**TABLE 3:** Functional outcomes of two groups.

	PMSA group	MNLA group	*p*
Patient satisfaction (*X ± S*)	9.56 ± 1.031	9.60 ± 0.737	0.904
VASSNP (*X ± S*)	0.13 ± 0.342	0.13 ± 0.352	0.948
ASS (*X ± S*)	10.94 ± 0.85	11.13 ± 0.35	0.428
NDI (*X ± S*)	0.81 ± 0.98	0.67 ± 0.98	0.678

Abbreviations: ASS, axial symptom scores; MNLA, midline nuchal ligament approach; NDI, neck disability index; PMSA, paramedian muscle‐splitting approach; VASSNP, visual analogue scale score for neck pain.

#### 3.1.3 Postoperative Complications

All surgical procedures were carried out successfully, with no instances of incision infection, vertebral artery injury, or dural tear. However, there was one patient with occipital numbness in the PMSA group, which was considered related to the traction injury of the greater occipital nerve (GON) during the exposure process; the symptom of numbness improved significantly through circulatory enhancement and Vitamin B12 supplementation at the postoperative 5th month.

### 3.2 Cadaver Study

**FIGURE 5:**
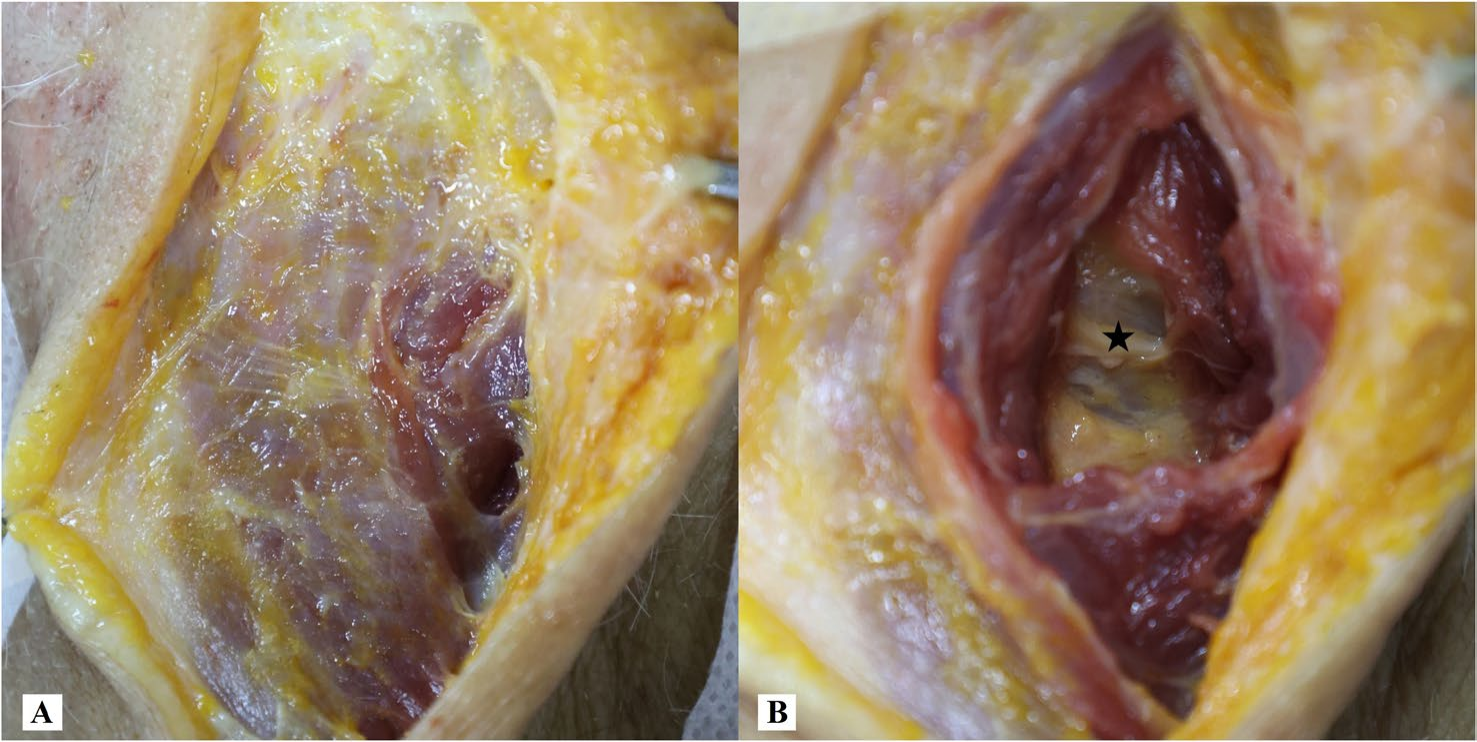
Posterior view of surgical exposure through the PMSA. (A) The exposure of the Traps and SpCa; (B) the blunt dissection of the SSCa, the black star indicates the GON.

**FIGURE 6:**
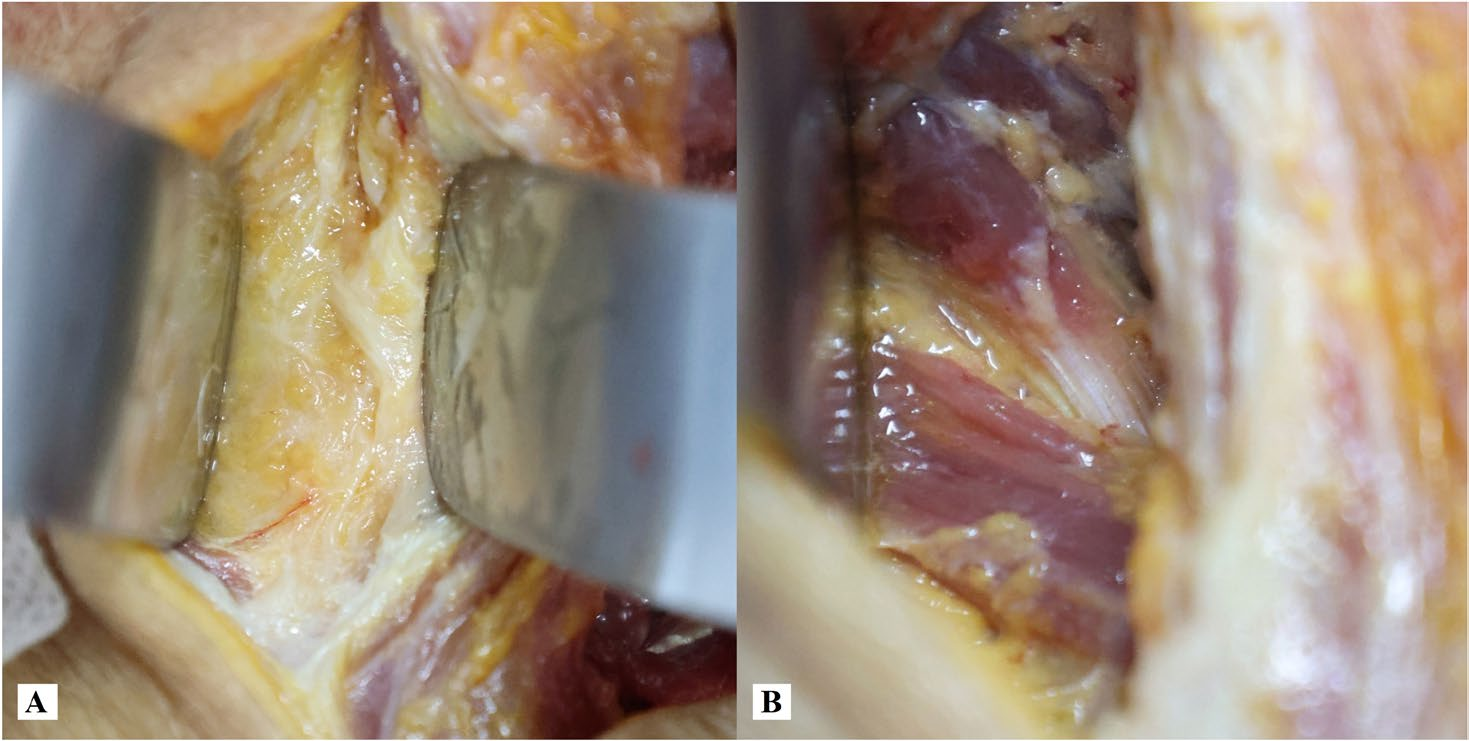
Posterior view of surgical exposure through the MNLA. (A) The ventral region of the nuchal ligament is occupied by loose connective tissue; (B) the exposure of the muscles connecting to the spinous process of C2, including the RCPma, OCI, and SSCe.

**FIGURE 7:**
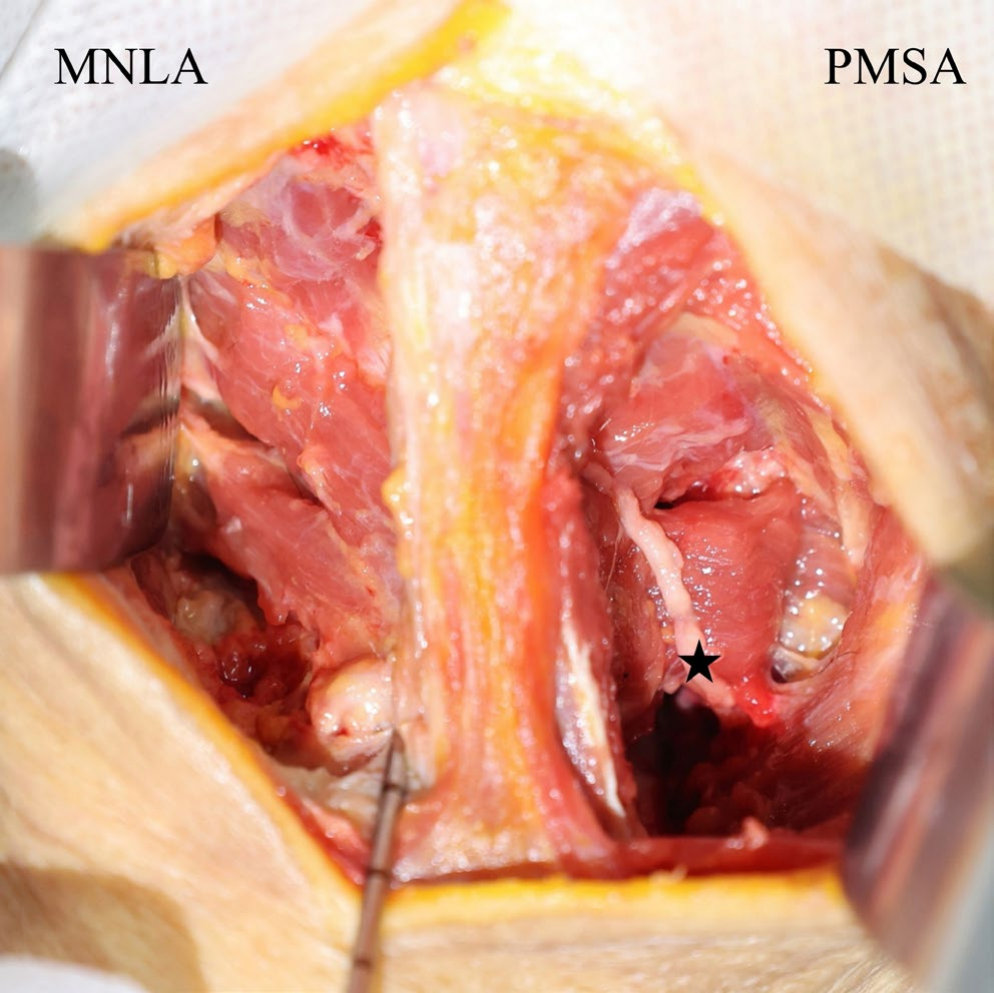
Cadaveric image following complete dissection after procedure of the MNLA and the PMSA. the black star indicates the GON.

A total of six fresh cadaveric specimens were successfully utilized for the comparative study. In cadaveric simulations of the PMSA, it was observed that the intersection between the Traps and SpCa exhibited significant anatomical variability. During blunt dissection of the SSCa to expose the RCPma, OCI and SSCe, the GON was found to be present within the surgical field in 2 of 6 specimens (Figures 5 and 7). However, in cadaveric simulations employing the MNLA, following the incision of the superficial nuchal ligament, the ventral region was observed to be occupied by loose connective tissue. Blunt dissection revealed the muscles connecting to the spinous process of C2, which included the RCPma, OCI and SSCe (Figures 6 and 7).

## 4 Discussion

Our findings revealed that the MNLA for minimally invasive temporary fixation of odontoid fractures may offer superior clinical outcomes, including shorter operative time, less intraoperative blood loss, and lower incidence of iatrogenic injury to the Traps, SpCa, SSCa, and GON when compared to the PMSA.

### 4.1 The Advantages and Current Status of Minimally Invasive Temporary Fixation for Odontoid Fractures

The traditional approach for temporary fixation of odontoid fractures requires disrupting muscle attachments on the midline structures and performing subperiosteal dissection of all posterior cervical muscles. In particular, the SSCe, RCPmi, RCPma, OCI are all compromised using this approach, leading to postoperative pain and marked dysfunction [8, 12, 21, 22, 23]. In contrast, the minimally invasive technique achieves a surgical procedure through the intermuscular planes, avoiding dissection of the posterior cervical muscles. This method maintains intact muscles with a robust blood supply, minimizes the amount of dead space, and reduces the incidence of deep wound infection, cerebrospinal fluid leakage, and postoperative pain [9, 22]. Furthermore, minimizing injuries to muscles and soft tissues results in reduced postoperative scar formation, which consequently diminishes the likelihood of the GON entrapment, postpones the degeneration of the atlantoaxial joint, and enhances neck function [6, 8, 22, 24, 25, 26, 27].

The minimally invasive technique has shown a lower occurrence of complications associated with procedures. Nevertheless, the clinical application of this method remains constrained due to the complex movements and anatomy of the atlantoaxial region. Research had shown that the dura mater was extensively connected to the RCPma, RCPmi, and OCI through a structure known as the myodural bridge [28, 29]. The connection plays a vital role in transmitting tensile forces during the contraction and relaxation cycles of the suboccipital muscles. It is essential for maintaining tension in the dura mater, facilitating the circulation of cerebrospinal fluid, and transmitting proprioceptive signals. Additionally, it had been reported that the force imbalance or dysfunction in these muscles serves as a key etiological factor in atlantoaxial degeneration [6]. Furthermore, the preservation of the SSCe, which attaches to the spinous process of C2, has shown statistically significant improvement in axial symptoms, decreasing the atrophy rate of muscles, and increasing the cervical range of motion [8]. Therefore, the two approaches employed in our research preserved the integrity of the inferior occipital musculature, aligning with modern minimally invasive surgical principles. Compared with the traditional open techniques, the two approaches reduced the iatrogenic injury and enhanced clinical outcomes.

### 4.2 Preservation of the OCI in Minimally Invasive Temporary Fixation

The OCI originates from the spinous process of C2 and inserts onto the transverse process of C1. Unilateral contraction induces contralateral head rotation, while bilateral contraction facilitates head extension. Additionally, studies have indicated that the GON may be constrained by the OCI, potentially resulting in occipital pain syndrome [30, 31]. However, the OCI hindered the placement of the titanium rods during minimally invasive temporary fixation for odontoid fractures (Figures 1H and 2H). Our research indicates that titanium rods can be effectively introduced using an insertion technique [32, 33] utilizing C2‐to‐C1 screw U‐channels. This approach helps to preserve the OCI and minimize the risk of injury to the C2 nerve and the C1–2 venous sinus during minimally invasive temporary fixation for odontoid fractures. We believe that this technology is feasible for the following reasons: (1) elimination of occipital bone obstruction and reduction of the risk of vertebral artery injury; (2) a space filled with loose connective tissue is located ventral to the OCI; (3) compared with the U‐channels of C1 lateral mass screws, the U‐channels of C2 pedicle screws are positioned closer to the ventral of the OCI, which makes it easy to accomplish the insertion of titanium rods; (4) the dimensions of the screw U‐channels provide a safe distance and protect the C1–C2 venous sinus and the C2 nerve root.

### 4.3 The Feasibility of the MNLA

The nuchal ligament is a triangular fibrous structure that stretches from the external occipital protuberance to the spinous process of C7, comprised of lamellar portion and funicular portion. The lamellar portion is composed of transverse fibers and may be associated with the trapezius tendon, whereas the funicular portion consists of loose connective tissue [34]. Additionally, it has been reported that posterior atlantoaxial surgery along the nuchal ligament can minimize muscle damage [35]. To evaluate the influence of the nuchal ligament on the functionality and stability of the upper cervical spine, we performed a radiographic analysis of 8074 cervical X‐rays obtained from our hospital prior to the clinical studies. Our findings indicated an absence of nuchal ligament calcification in the upper cervical spine. Additionally, we assessed 689 patients with upper cervical injuries through sagittal T2‐weighted and STIR sequences of MRI. This evaluation did not reveal any characteristics indicative of the nuchal ligamentous tears at the upper cervical spine. The findings suggest the nuchal ligament at the upper cervical spine may have no effect on the functionality and stability of the upper cervical spine.

### 4.4 The Advantages of the MNLA

The MNLA for temporary fixation of odontoid fractures demonstrated distinct advantages in shortening operative time, reducing postoperative edema of cervical posterior muscles and minimizing GON interference. Its surgical procedure was similar to the open approach, using the spinous process of C2 and the OCI as anatomical landmarks, which provide a constant and clear surgical field. Critically, the exposure of the spinous process of C2 in this approach facilitated the fixation of navigation reference markers, thereby enabling an ideal combination of this approach with intraoperative navigation, which enhanced the safety and accuracy of screw placement during atlantoaxial minimally invasive surgery [7]. Moreover, this approach could be extended to complex procedures such as spinal canal decompression and intramedullary tumor resection [36].

### 4.5 The Advantage and Disadvantages PMSA

The PMSA usually used the trapezius‐splenius capitis interface as the anatomical landmark [11, 13] and retained the intact nuchal ligament. However, our anatomical study revealed significant variability in the trapezius‐splenius capitis interface, which strongly correlated with trapezius muscle variations [37], the anatomical heterogeneity increased surgical exposure complexity. To address this problem and safely perform the PMSA, some researchers proposed that the Traps and SpCa were dissected at a consistent distance from the midline, generally 1.5 cm lateral to the spinous process of C2 [22]. However, this modified method increased the iatrogenic damage of Traps and SpCa.

The GON usually originates from the posterior branch of the C2 spinal nerve. It travels through the intermuscular plane located between the OCI and SSCa, subsequently passing through the SSCa and Traps to innervate the skin of the occipitocervical region. However, our anatomical study revealed that in 2 of 6 specimens, the GON penetrated the SSCa close to the median line, causing it to transverse the surgical field (Figures 5B and 7), which was consistent with a previous report on the course of the GON [38]. Furthermore, in our clinical study, a patient who underwent the PMSA showed symptoms of the GON traction injury, mainly manifested as numbness and pain in the occipital region, and the above symptoms gradually improved significantly at the postoperative 6th month. Therefore, before performing the PMSA, we recommend that three‐dimensional MRI of the GON [39] be performed to delineate its precise anatomical course, which may reduce the risk of iatrogenic injury during the surgery.

### 4.6 Limitations and Prospect of This Research

It is important to recognize that the occurrence of this fracture was infrequent in clinical settings and its relatively small sample size was limited. Additionally, our study is limited by the constraints of a single‐center retrospective design, the differences between anatomical studies and clinical applications, and the lack of evaluation regarding the mechanisms underlying muscle injury. So, it is essential to increase the sample size and extend the follow‐up duration to further assess the long‐term effectiveness of the two approaches in the future, thereby providing more reliable reference data for clinical practice.

## 5 Conclusions

This study has demonstrated that both the MNLA and the PMSA can achieve favorable clinical outcomes in the treatment of odontoid fractures. However, compared with the PMSA, the MNLA, using the spinous process of C2 and the OCI as anatomical landmarks, offers advantages of the stability of the surgical procedure, easy exposure, and reduced iatrogenic damage to the cervical posterior muscles and GON.

## Author Contributions

Conceptualization: Youcai Qiu and Liang Wang. Data curation: Yang Li and Yijin Wang. Formal analysis: Yang Li and Yijin Wang. Funding acquisition: Xuhua Lu. Investigation: Youcai Qiu and Liang Wang. Methodology: Youcai Qiu and Xuhua Lu. Resources: Liang Wang and Xuhua Lu. Supervision: Xuhua Lu. Writing – Original Draft: Youcai Qiu and Yijin Wang. Writing – review and editing: Youcai Qiu and Liang Wang.

## Funding

This work was supported by the fund of the Shanghai Oriental Talents Top‐Tier Program (No. SHSDFYCBJ‐LXH).

## Ethics Statement

All procedures performed in studies involving human participants were in accordance with the ethical standards of the Ethics Committee of Naval Medical University and with the 1964 Helsinki declaration and its later amendments or comparable ethical standards. Written informed consent was obtained from all individual participants included in the study.

## Conflicts of Interest

The authors declare no conflicts of interest.

## Data Availability

The data that support the findings of this study are available from the corresponding author upon reasonable request.
